# Time to diagnostic certainty for saddle pulmonary embolism in hospitalized patients

**DOI:** 10.17305/bb.2022.8393

**Published:** 2023-08-01

**Authors:** Yuliya Pinevich, Amelia K Barwise, John Matthew Austin, Jalal Soleimani, Svetlana Herasevich, Sarah Redmond, Yue Dong, Vitaly Herasevich, Ognjen Gajic, Brian W Pickering

**Affiliations:** 1 Department of Anesthesiology and Perioperative Medicine, Mayo Clinic, Rochester, MN, USA; 2 Division of Pulmonary and Critical Care Medicine, Mayo Clinic, Rochester, MN, USA; 3 The Johns Hopkins University School of Medicine, Baltimore, MD, USA; 4 Kern Center for the Science of Health Care Delivery, Mayo Clinic, Rochester, MN, USA

**Keywords:** Pulmonary embolism (PE), performance measure, time to diagnosis, diagnostic error, diagnostic delay, electronic health records

## Abstract

There is a lack of diagnostic performance measures associated with pulmonary embolism (PE). We aimed to explore the concept of the time to diagnostic certainty, which we defined as the time interval that elapses between first presentation of a patient to a confirmed PE diagnosis with computed tomography pulmonary angiogram (CT PA). This approach could be used to highlight variability in health system diagnostic performance and to select patient outliers for structured chart review in order to identify underlying contributors to diagnostic error or delay. We performed a retrospective observational study at academic medical centers and associated community-based hospitals in one health system, examining randomly selected adult patients admitted to study sites with a diagnosis of acute saddle PE. One hundred patients were randomly selected from 340 patients discharged with saddle PE. Twenty-four patients were excluded. Among the 76 included patients, time to diagnostic certainty ranged from 1.5 to 310 hours. We found that 73/76 patients were considered to have PE present on admission (CT PA ≤ 48 hours). The proportion of patients with PE present on admission with time to diagnostic certainty of > 6 hours was 26% (19/73). The median (IQR) time to treatment (thrombolytics/anticoagulants) was 3.5 (2.5–5.1) hours among the 73 patients. The proportion of patients with PE present on admission with treatment delays of > 6 hours was 16% (12/73). Three patients acquired PE during hospitalization (CT PA > 48 hours). In this study, we developed and successfully tested the concept of time to diagnostic certainty for saddle PE.

## Introduction

Pulmonary embolism (PE) is considered a frequently missed medical condition and a major cause of preventable hospital death. Pulmonary emboli may present with a range of non-specific symptoms, which can be easily dismissed in patients with comorbidities [1]. One multicenter qualitative study that explored physician-reported errors in diverse clinical settings found that PE was reported to be the most prevalent medical misdiagnosis, with nearly half of cases related to failure or delay in considering the diagnosis of PE [2]. A systematic review and meta-analysis estimated that 10% of harmful diagnostic errors in hospitalized patients were related to missed or delayed PE, which was the second most common missed diagnosis [3]. Several studies describing autopsies noted that 55%–70% of PE diagnoses were missed prior to death [4–8]. An analysis of claims in malpractice litigation found that PE is a common basis for death lawsuits, with 62% classified as an allegation of “failure to diagnose and treat” [9].

While there is substantial evidence of underdiagnosis, overdiagnosis of PE is also common [10]. Epidemiologic studies show an upward trend in PE incidence in parallel with stable or decreasing mortality, which may result from improved identification of PE, in particular subsegmental variants [11–15]. In some circumstances, it may be beneficial to leave some types of PE, such as isolated subsegmental PE, untreated [16]. Overdiagnosis of PE can cause iatrogenic harm and increase costs [17, 18]. Given that PE is frequently misdiagnosed, it is important to assess diagnostic fidelity for PE at the institutional level.

The existing approaches to operationally define and measure missed or delayed diagnoses are largely ineffective [18, 19]. One specific challenge is the lack of valid and reliable performance measures for PE. A literature review and exploration of the National Quality Forum’s measure database did not identify any currently endorsed measures that assess hospital performance related to the diagnostic process for PE.

Structured chart reviews are widely used to measure diagnostic performance, despite time- and labor-intense procedures [20]. Several e-triggers, such as emergency department/primary care visits followed by unplanned hospitalization, readmission, unexpected intensive care unit (ICU) admission, and abnormal test result without timely follow-up, were previously proposed for efficient selection of high-risk patients [20–22]. However, these electronic health record (EHR) signals are too general to be applied to the identification of patient outliers for structured chart review focused on the PE diagnostic performance of a health system.

In this study, we aimed to explore the concept of the time to diagnostic certainty (TDx), which we defined as the duration of time that elapses between the first presentation of a patient to the moment of a definitive documented diagnosis of pulmonary saddle embolism. We hypothesized that time to diagnostic certainty could serve as a reliable, easily deployed measure to highlight variability in diagnostic performance of a health system; and to identify patient outliers for structured chart review focused on the identification of the underlying contributors to diagnostic error or delay. For this proof of concept study, we limited our diagnosis search to saddle PE variants as they are more likely to present as massive or submassive PE [23]. It has been shown that saddle PE is associated with high rates of cardiac arrest, and cardiac and respiratory failure than other PE variants and therefore may have a substantial negative impact on patient outcomes [24].

## Materials and methods

### Study design and setting

This was a retrospective observational study of patients admitted with saddle PE to academic medical centers and associated community-based hospitals in the Mayo Clinic Health System. Mayo Clinic in Rochester, MN, USA, is an academic medical center with 62,000 annual hospital admissions and 14,800 annual ICU admissions. Mayo Clinic, Jacksonville, FL, USA, is an academic hospital that has 15,000 inpatient admissions and 3600 ICU admissions per year. The third academic medical center, Mayo Clinic, Phoenix, AZ, USA, has more than 12,000 inpatient admissions and 1800 ICU admissions per year. Overall, the Mayo Clinic health system has more than 120,000 inpatient admissions per year, including more than 600 annual PE cases as identified through ICD diagnosis codes.

### Population

We included patients ≥18 years of age admitted to the study sites from 1 June 2018 to 31 December 2020 with the diagnosis of acute saddle PE and provided research authorization [25]. Saddle PE is a large pulmonary embolism that straddles the bifurcation of the pulmonary trunk, extending into the left and right pulmonary arteries. We excluded patients with other variants of PEs, patient transfers from non-Mayo facilities, elective admissions, and those receiving comfort care. Patients that were transferred within Mayo Clinic Health System were included.

### Definition of pulmonary embolism

To test the principle that TDx is a useful measure of diagnostic performance, a gold standard diagnosis of PE was defined as PE confirmed on a computed tomography pulmonary angiography (CT PA) scan. We did not accept alternative diagnostic evidence such as cardiac echocardiography; or diagnostic suspicion documented in the physician note due to concern about variability in the availability and interpretation of these alternatives.

### Data collection and EHR review

The population of interest was extracted using Advanced Text Explorer (ATE). ATE is a web-based tool that allows text search in all clinical notes, as well as radiology, pathology, and lab reports. We used search queries of discharge notes for the diagnosis of acute saddle PE. The queries included a search for multiple words in a quoted phrase within 10 words from each other (∼10). The queries were “Saddle PE” or “Saddle Pulmonary Emboli”∼10 or “Saddle Pulmonary Embolus”∼10 or “Saddle Pulmonary Embolism”∼10 not “History of pulmonary embolism”∼10 in discharge summary notes. We used simple randomization in JMP Pro 14.1.0 software (SAS Institute Inc., Cary, NC, USA) to create a random sample for an EHR review. A physician–researcher with clinical and research expertise in the domain of acute care (YP) performed the EHR review of 100 patients. The following data points were extracted: patient age, sex, hospital and floor location, admission source, time of first presentation (T0), the time when the result of CT PA was posted in the EHR, the time thrombolytics or anticoagulants were initiated at a therapeutic dose, time of hospital and ICU admission and discharge. Charlson Comorbidity Index, a validated method to assess patient comorbidity, was calculated based on electronic note search strategies, as described elsewhere [26]. A physician–researcher (YP) and a PhD researcher with qualitative and mixed-methods research expertise in the field of diagnostic errors (SR) [27] reviewed EHR in order to assess possible reasons for diagnostic delays in selected patients.

### Outcome measurements

Time to diagnosis studies refer to studies that evaluate the interval from time of first presentation (T0), which is the time of first alert symptoms or the time of first medical contact, to the time of diagnosis [28]. For the present study, T0 was defined as the time of presentation to the emergency room or outpatient clinic (<12 hours before hospital admission), or time of admission to the hospital, whichever came first. A group of critical care experts determined that for the purpose of our study, diagnostic certainty was achieved when the result of the CT PA was documented in the EHR. TDx was the time that elapsed between T0 and the time the result of CT PA was reported in the EHR.

Based on the local expert review and our assessment of the literature, a TDx of 6 hours was chosen as a reasonable target time for a diagnosis of symptomatic PE to be reached in hospitalized patients [29–33]. That is a time interval that was considered sufficient for diagnosis to be made in acute care settings in our particular healthcare system [34]. The primary outcome of interest was the proportion of patients, with a saddle PE present on admission with TDx of greater than 6 hours.

**Figure 1.**
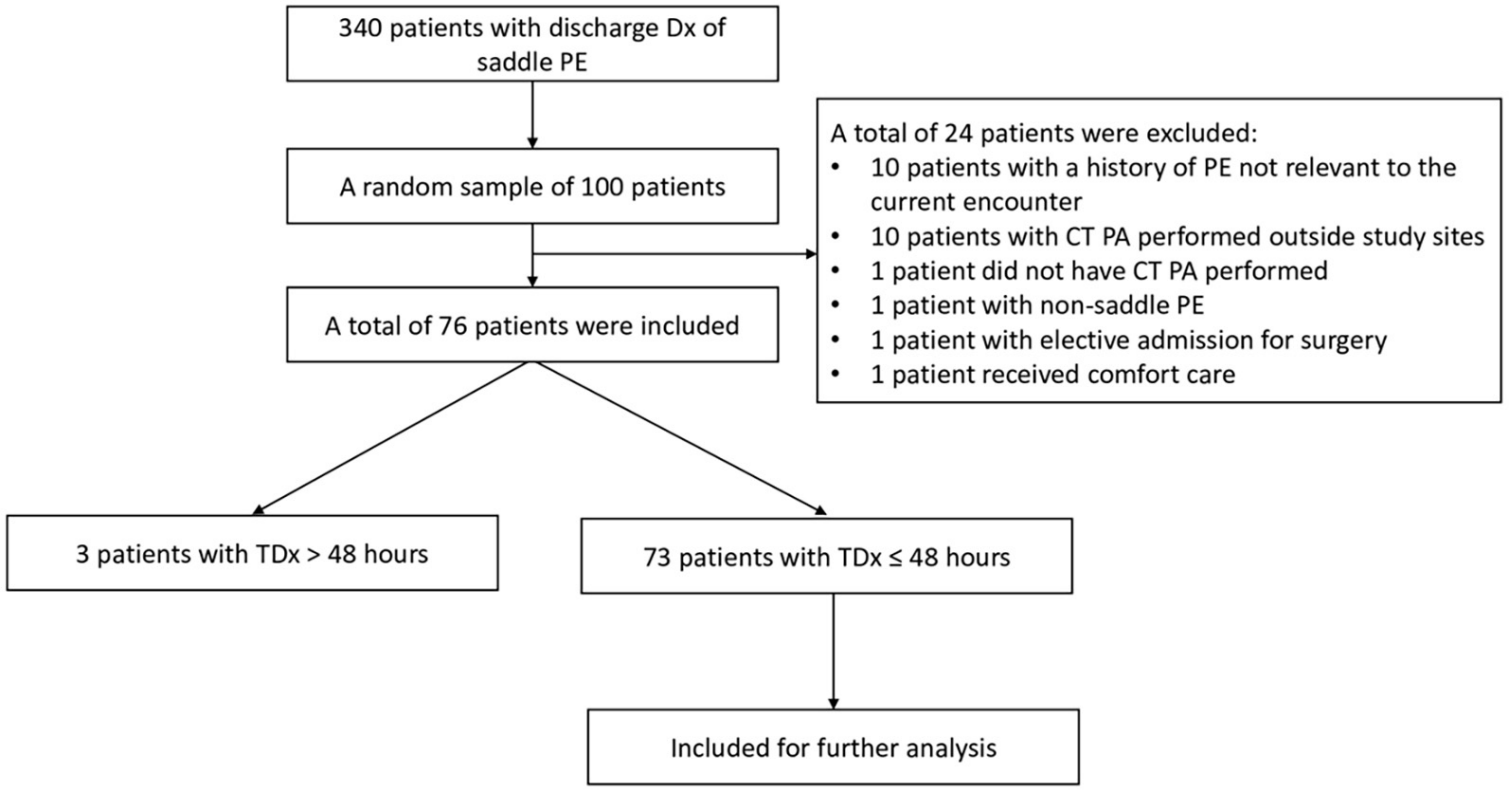
**Flowchart diagram.** CT PA: Computed tomography pulmonary angiography; Dx: Diagnosis; PE: Pulmonary embolism; TDx: Time to diagnostic certainty; TDx ≤ 48 hours: PE present on admission; TDx > 48 hours: PE acquired during hospitalization.

Secondary outcomes of interest included time to treatment (TTx) of PE, the proportion of patients with treatment delays of greater than 6 hours, and the number of patients with hospital-acquired PE. Time of treatment was the time of administration of thrombolytics or anticoagulants at a therapeutic dose as documented in the medication administration record. TTx was the time that elapsed between T0, as defined earlier, and the time of treatment. The treatment time that exceeded 6 hours was classified as a treatment delay. Patients were categorized as those with PE acquired during hospitalization when TDx exceeded 48 hours.

### Reliability

To ensure reliability of data extraction and TDx calculation, a second physician–researcher (JS) with an extensive experience in chart reviews, reviewed the EHR using a standard operating procedure (SOP) extracted the T0 and the time when the result of CT PA was posted in the health record. The TDx was calculated independently from the first reviewer. Inter-rater reliability of TDx between two reviewers was assessed using Kappa (K) agreement statistics.

### Ethical statement

The Mayo Clinic Institutional Review Board (Rochester, MN, USA) approved the study as minimal risk (18-007115). The requirement for informed consent was waived following institutional review.

### Statistical analysis

Descriptive statistics and analysis were done using JMP Pro 14.1.0 software (SAS Institute Inc., Cary, NC, USA). Categorical variables were reported as counts with percentages and analyzed with Fisher’s exact test. Continuous variables were reported as medians with interquartile range (IQR) and analyzed with Wilcoxon’s rank-sum test. Inter-rater reliability was assessed with K agreement statistics [34]. A two-sided *P* value of less than 0.05 was considered statistically significant.

## Results

### Patient cohort identification

The ATE search resulted in 437 discharge notes posted in EHR and 340 unique patients with a discharge diagnosis of saddle PE. A total of 100 patients were randomly selected for EHR review (Figure 1). A total of 21 patients were excluded as acute PE was not confirmed within the designated time frame, such as history of PE that was not relevant to the encounter included in our study (*n* ═ 10), CT PA performed outside study sites (*n* ═ 10), CT PA was not done (*n* ═ 1) (Figure 1). Other exclusions were due to the following reasons (*n* ═ 3): presence of non-saddle PE, comfort care order measures in place, and elective admission for surgical procedure. Therefore, a total of 76 patients were included for detailed EHR review. The median age in this cohort was 72.5 years (IQR 66–82). A total of 33/76 (43%) were female.

### Time to diagnostic certainty

TDx for PE was calculated for each patient encounter and ranged from 1.5 to 310 hours. If TDx exceeded 48 hours, the chart was reviewed for hospital-acquired PE. Three of 76 patients (3.9%) had a diagnosis of PE made > 48 hours after admission (Figure 1). Following the chart review, all three cases were considered to be acquired with no signs of PE on admission (Table 1).

**Table 1:** Patients with hospital-acquired pulmonary embolism

**N**	**Patient history**	**Case description**	**TDx (h)**	**TTx (h)**
1	68 yo, Male. History of hypertension and DM type 2	∙ Patient admitted with traumatic subarachnoid hemorrhage, subdural hematoma, and intraparenchymal hemorrhage secondary to motor vehicle accident. ∙ CT PA on admission to exclude thoracic injury, did not show PE. ∙ Day 12 AMS and hypotension. Echo showed evidence of right heart strain. ∙ Day 13 CT PA performed, low intensity anticoagulation initiated, and an IVC filter placed.	311	310
2	60 yo. Male. UC, post total colectomy, ileostomy, multiple episodes of intestinal obstruction.	∙ Intestinal obstruction/volvulus on admission, the exploratory laparotomy with adhesiolysis. ∙ Day 10 CT abdomen with IV contrast performed (partial visualization of PE), followed by CT PA.	280	281
3	83 yo, Male. CAD with UA; prostate cancer, pelvic mass with bilateral obstruction and hydronephrosis and bilateral lower extremity edema secondary to venous obstruction.	∙ Chief complain of shortness of breath and peripheral edema that was thought to be related to a large pelvic mass. ∙ Day 1 CT PA did not show PE. ∙ Day 4 bilateral nephrostomy tubes placed due to hydronephrosis. ∙ Day 5 NSTEMI identified, two drug-eluting stents placed. ∙ Day 9 a syncopal event, followed by CT PA: PE confirmed.	214	224

AMS: Altered mental status; CAD: Coronary artery disease; CT: Computed tomography; CT PA: Computed tomography pulmonary angiography; DM: Diabetes mellitus; Echo: Echocardiography; h: Hours; IV: Intravenous; IVC: Inferior vena cava; NSTEMI: Non-ST segment elevation myocardial infarction; PE: Pulmonary embolism; TDx: Time to diagnostic certainty; TTx: Time to treatment; UA: Unstable angina; UC: Ulcerative colitis; yo: Years of age.

**Figure 2.**
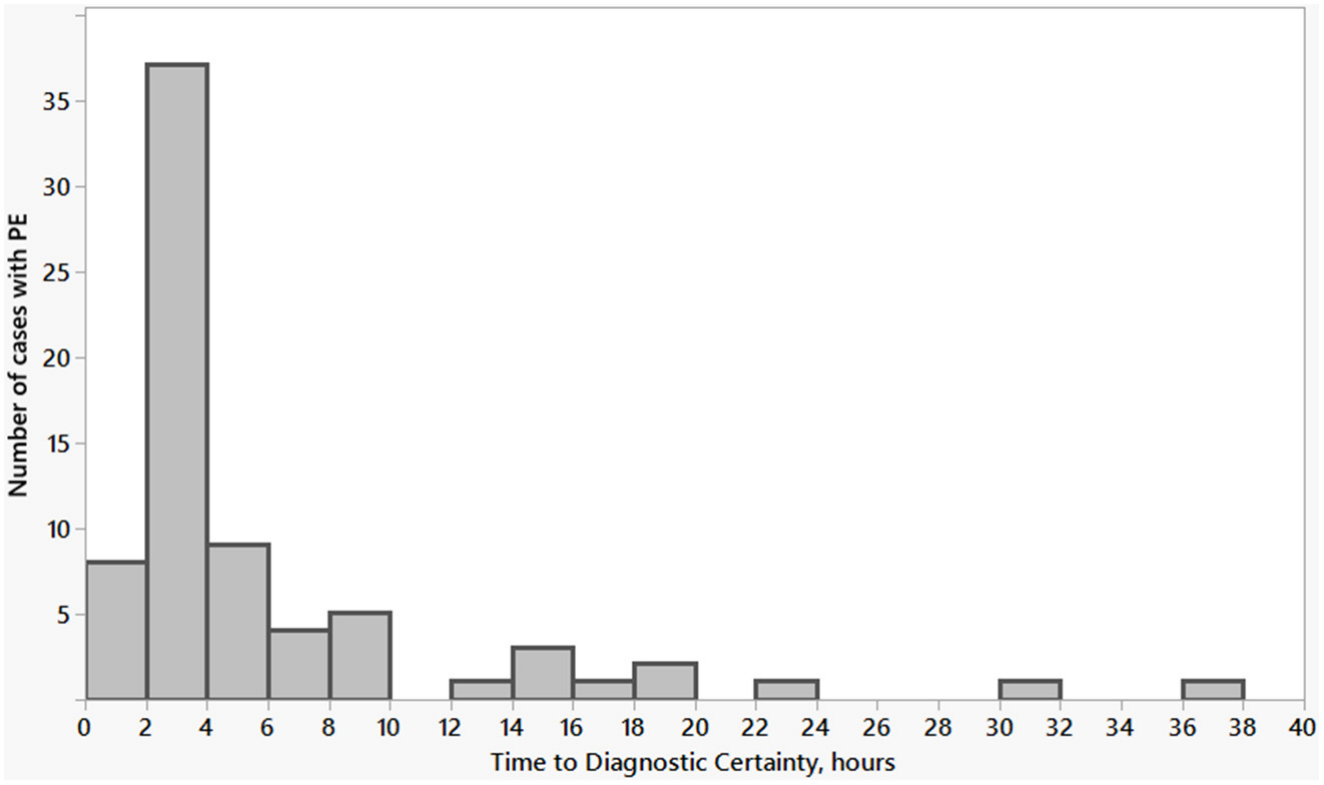
**Time to diagnostic certainty for pulmonary embolism present on admission, hours.** Analysis of the subgroup of patients with PE present on admission determined the median time to diagnostic certainty for PE was 3.4 (IQR 2.6–6.3) hours. Nineteen of 73 (26%) patients reached diagnostic certainty outside 6-hour time period. PE: Pulmonary embolism.

Of the 76 patients, 73 had saddle PE present on admission and were included in further analysis. The median TDx was 3.4 (IQR 2.6–6.3) hours (Figure 2). Among those who had a diagnosis of PE on admission, 19 patients (26%) had TDx greater than 6 hours. Characteristics of patients with PE present on admissions are described in Table 2. There was no significant difference in age, sex, and comorbidities between patients with TDx > 6 hours and those with TDx ≤ 6 hours. The frequency of CT PA performed after transfer from community hospital to academic hospital was not significantly different between the groups. Patients with prolonged TDx for PE (>6 hours) were more likely to have a diagnosis of PE established during the ICU stay. Hospital and ICU length of stay, in-hospital mortality were not significantly different between two groups.

**Table 2:** Characteristics of patients with pulmonary embolism present on admission

**Variable**	**All patients with PE present on admission, *N* ═ 73**	**Patients with TDx > 6 hours, *N* ═ 19**	**Patients with TDx ≤ 6 hours, *N* ═ 54**	***P* value**
Age, years	73 (66–82)	77 (62–83)	77.5 (65.8–84.0)	0.37^a^
Sex, female	33 (45.2)	9 (47.4)	24 (25)	0.83^b^
Charlson Comorbidity Index	6 (5–9)	6 (4–9)	6 (5–10)	0.58^a^
CT PA performed after transfer	11 (24.6)	7 (36.8)	4 (7.4)	0.06^b^
CT PA performed during ICU stay	12 (16.4)	9 (47.4)	3 (5.5)	<0.0001^b, c^
ICU LOS, days	1.6 (0.9–2.1)^d^	1.3 (0.6–2.0)^e^	1.6 (1.0–2.1)^f^	0.33^a^
Hospital LOS, days	4.2 (2.6–6.2)	3.9 (2.1–5.5)	4.0 (2.8–5.6)	0.39^a^
TDx, hours	3.4 (2.6–6.3)	12.6 (8.4–18.2)	3.1 (2.2–3.5)	<0.0001^a, c^
TTx, hours	3.5 (2.5–5.1)	5.9 (3.5–10.3)	3.1 (2.2–4.2)	0.0001^a,c^
In-hospital mortality	3 (4.1)	2 (10.5)	1 (1.8)	0.16^b^

Numbers indicate *N* (%) and median (IQR). ^a^Wilcoxon’s rank-sum test; ^b^Fisher exact test; ^c^Significant; ^d^*N* ═ 51; ^e^*N* ═12; ^f^*N* ═ 39. CT PA: Computed tomography pulmonary angiogram; ICU: Intensive care unit; LOS: Length of stay; PE: Pulmonary embolism; TDx: Time to diagnostic certainty; TTx: Time to treatment.

**Figure 3.**
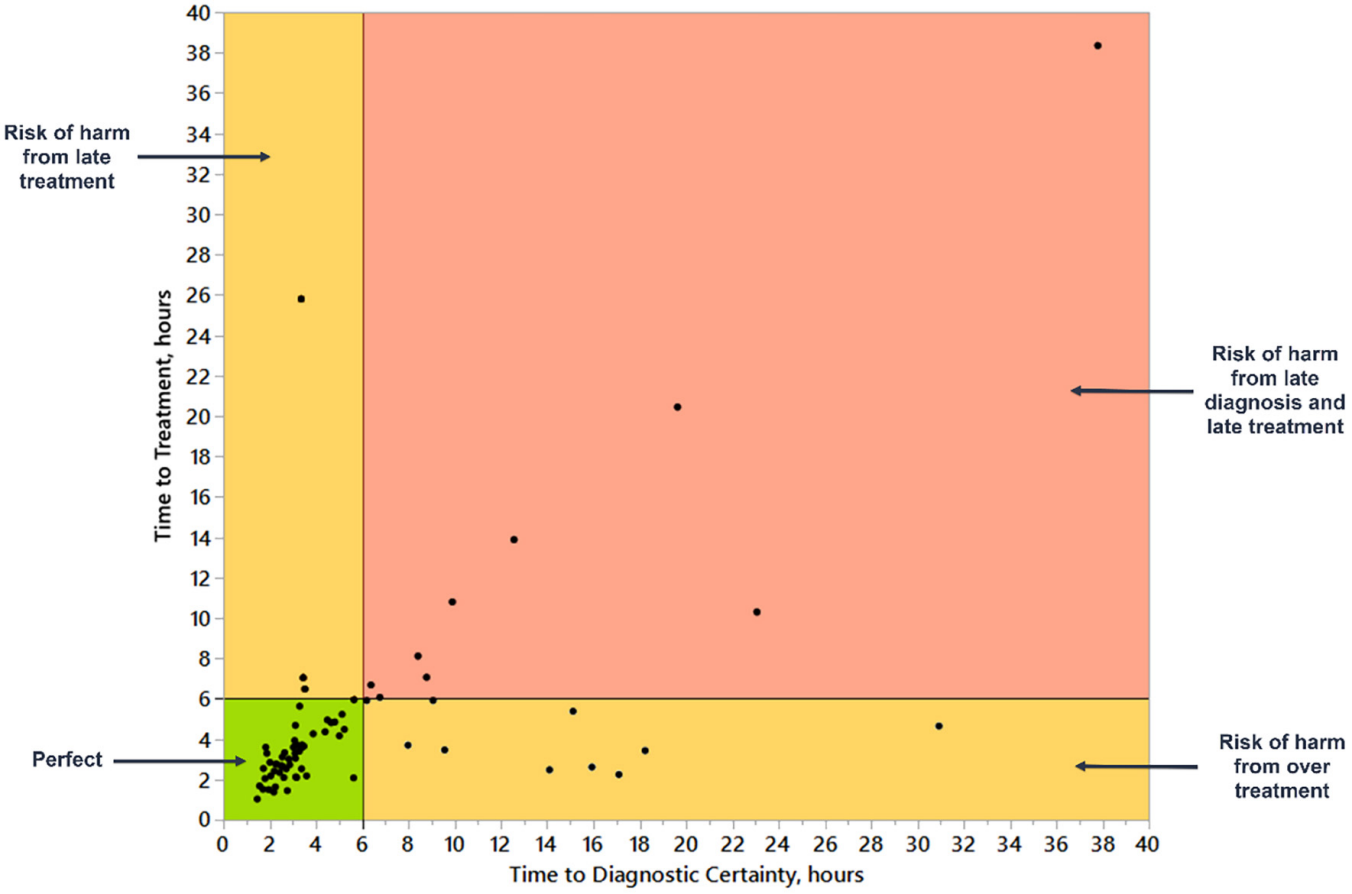
**Bivariate analysis of time to diagnostic certainty and time to treatment, hours.** Figure describes the relationship between time to diagnostic certainty and time to treatment in the 73 patients with pulmonary embolism present on admission. Based on time to diagnostic certainty and time to treatment, there were four groups of patients: 1) those with perfect diagnostic performance (early diagnosis and treatment), 2) risk of harm from late treatment (early diagnosis and late treatment), 3) risk of harm from overtreatment (early treatment and late diagnosis), and 4) poor diagnostic performance (risk of harm from late diagnosis and treatment).

### Time to treatment

TTx ranged from 1 hour to 310 hours. In the 73 patients with PE present on admission, the median TTx was 3.5 (2.5–5.1) hours. The proportion of patients with PE present on admission with treatment delays of > 6 hours was 16% (12/73). TTx was significantly higher in the group of patients with TDx of greater than 6 hours (Table 2).

We evaluated the relationship between TDx and TTx in the 73 patients with PE present on admission (Figure 3). Most patients (*n* ═ 51, 69.9%) had both diagnosis and treatment within 6-hour period (perfect diagnostic performance). Some patients (*n* ═ 10, 13.7%) got treatment before diagnosis (risk of harm of overtreatment). Some patients (*n* ═ 3, 4.1%) received diagnosis within a 6-hour interval, but their treatment was delayed. Some patients (*n* ═ 9, 12.3%) that received both diagnosis and treatment outside a 6-hour interval, were the group of most interest for structured chart review to identify underlying contributors to diagnostic error or delay.

**Table 3:** Contributors to diagnostic error and delay in selected patients

**N**	**Patient history**	**Case description**	**TDx (h)**	**TTx (h)**	**Possible cause of delay**
1	77 yo, F. Dementia, epilepsy, EHT, DM type 2.	∙ Syncope at home; community hospital ED visit. ∙ Academic hospital admission (within 1 hour): hypotension and vasopressor support. ∙ Suspected problems were hypovolemic shock, sepsis. ∙ DEOD of PE with heart right strain. ∙ Doppler US subsequently revealed DVT of the left leg.	37.8	38.4	**Patient factors**: dementia, nonverbal at baseline ^a^, debilitat ion, multiple falls related to syncopal episodes prior to the latest syncope^b^. **Cognitive factors:** premature “narrowing in” on hypovolemic shock and sepsis diagnosis, failure to consider cardiogenic shock due to PE on differential list^c^. **Provider factors**^d^.
2	84 yo, F. Graves disease, recently diagnosed uncontrolled hyperthyroidism and normal pressure hydrocephalus.	∙ Previous hospitalization due to new left wrist fracture, metabolic encephalopathy. ∙ 7-days readmission: AMS and new oxygen requirements. ∙ US ordered due to edema of the left leg, showed an obstructive popliteal artery thrombus, but no DVT. ∙ Heparin administered to treat arterial thrombosis. ∙ The CT PA postponed.	23	10.3	**Patient factors**: AMS^a^. **Cognitive factors:** risk of harm greater than benefit -risk of excessive contrast dye administration for peripheral angiography and CT PA^e^.
3	79 yo, M. Ischemic cardiomyopathy, CHF; chronic UTIs, recent hospitalization for urosepsis/pyelonephritis, nephrostomy tube.	∙ Admission due to displacement of nephrostomy catheter. ∙ New oxygen requirements and abnormal CT chest (new patchy/nodular left lower lobe consolidative and ground glass opacities with bilateral bronchial wall thickening) were initially considered as CAP. ∙ The next morning: CT was re-interpreted by radiologists: PE was suggested (heterogeneous appearance of the pulmonary arteries with some increased density posteriorly) and further confirmed by CT PA (large saddle pulmonary embolus with extension into the lobar and segmental arteries of the bilateral upper and lower lobes. Right ventricular dilatation, mild bowing of the interventricular septum and reflux of contrast into the IVC compatible with right heart strain). ∙ Antibiotics discontinued.	19.6	20.5	**Patient factors**^b^. **Cognitive factors:** premature ‘narrowing in’ a diagnosis of pneumonia^c^, failure to order an appropriate test (CT PA)^e^. **Provider factors:** an experienced radiologist revised the findings of native chest CT in 7.3 hours after the results posted; and facilitated CT PA order^d,f^.
4	88 yo, F. Dementia, EHT, bronchiectasis.	∙ 5-days readmission after COVID-19-hospitalization. ∙ New onset of dyspnea was initially explained by possible aspiration/bacterial pneumonia (the diagnosis was excluded). ∙ CT PA ordered at ED department. ∙ Antibiotics discontinued.	12.6	13.9	**Patient factors**: dementia, AMS^a^. **System and Process factors**: time from provider order to CT PA result posted in EHR was 7.7 hours^g^.
5	73 yo, M. Obesity and CAD.	∙ Known COVID-19-associated pneumonia. ∙ PE and DVT workup was triggered by high level of D-dimers and right leg swelling.	9.9	10.8	**Organization and Infrastructure factors**: the difference between time of check in at ED and time of clinician note/lab order was 7 hours^h^.
6	53 yo, M. EHT, DM type 2 and past alcohol abuse.	∙ Dyspnea and syncope before hospitalization ∙ ED: hypoxia and tachycardia ∙ US of low extremities confirmed DVT ∙ CT PA results followed by mechanical thrombectomy of bilateral PE: hypoxemia leading to PEA and CA with unsuccessful resuscitation.	8.7	7	**Cognitive factors:** risk of harm greater than benefit - CT PA was initially postponed due to high creatinine^e^.
7	86 yo, F. CAD, DM type 2, hiatal hernia with GERD, s/p Nissen fundoplication.	∙ Shortness of breath during one week before hospitalization ∙ CAP and PE on differential list. ∙ CT PA ordered within ED stay.	8.4	8.1	**System and Process factors**: patient was initially seen at outpatient visit, then sent to ED^i^.
8	70 yo, M. BPH status post PVP, CKD, and recurring UTIs.	∙ Prior treatment of urinary retention and UTI. ∙ 4-days readmission: bilateral calf pain, fever and fatigue. ∙ The DVT and PE were considered at the ED.	6.8	6.1	**System and Process factors:** patient was initially seen at outpatient visit, then sent to ED^i^.
9	67 yo, M. EHT, MDD, COPD.	∙ PE and left lower extremity DVTs diagnosed at ED	6.4	6.7	The factors contributing to DEOD are uncertain.

AMS: Altered mental status; BPH: Benign prostatic hyperplasia; CA: Cardiac arrest; CAD: Coronary artery disease; CAP: Community-acquired pneumonia; COPD: Chronic obstructive pulmonary; COVID-19: Coronavirus disease; CKD: Chronic kidney disease; CT PA: Computed tomography pulmonary angiography; DEOD: Diagnostic error or delay; DVT: Deep vein thrombosis; DM: Diabetes mellitus; Dx: Diagnosis; ED: Emergency department; h: Hours; MDD: Major depressive disorder; EHT: Essential hypertension; F: Female; GERD: Gastroesophageal reflux disease; M: Male; PE: Pulmonary embolism; PEA: Pulseless electrical activity; PVP: Photoselective vaporization of the prostate; TDx: Time to diagnostic certainty; TTx: Time to treatment; UTI: Urinary tract infection; US: Ultrasound. **Contributing factors: **^a^challenges in communicating with patients due to their intellectual capacity, language, or cooperativeness; ^b^high medical complexity that confuses the diagnostic process; ^c^premature ‘narrowing in’ or ‘anchoring’ on a specific diagnosis for diverse reasons; ^d^failure to engage ‘new set of eyes’ or colleagues with different expertise or experience in timely fashion, due to a variety of reasons; ^e^an over-reliance on the ‘truth’ of diagnostic tests; ^f^limited clinical experience of key care team members; ^g^delays in obtaining key lab results; ^h^physical location of patients in hospital or on unit that makes them relatively inaccessible to the care team; ^i^needing to go through many people or a chain of command in order to get things done.

Table 3 summarizes cases with prolonged TDx accompanied by treatment delays of > 6 hours. In order to assess possible reasons for diagnostic delays, we applied the sociotechnical framework previously used in our other studies to understand diagnostic error or delay [35]. Based on knowledge gained during that work, two reviewers (YP, SR) identified the following contributing categories as most frequently causing diagnostic error or delay in acute care settings: Care Team Interactions, Systems and Process factors, Patient, Provider, and Cognitive factors, and Organization and Infrastructure factors.

### Reliability

Inter-rater reliability of identifying TDx was measured. Agreement between two physician–researcher reviewers occurred in 74/76 cases. The inter-rater reliability coefficient (Kappa) for TDx was 0.97 (95% CI 0.94–1.0). This is almost perfect degree of agreement.

## Discussion

We performed a retrospective observational study of hospitalized patients with acute saddle PE and confirmed that TDx can be reliably abstracted from the EHR (Kappa value > 0.9). TDx based on readily identifiable EHR data is amenable to automation.

TDx allows us to highlight variability in diagnostic performance within a health system. In our study the median TDx for acute saddle PE present on admission was 3.4 hours. Similar median time was reported in the retrospective study that calculated time to PE diagnosis measured as the time interval between patient admission at the emergency department and CT PA examination [29]. More than a quarter of our study patients (19/73) were identified as having TDx that exceeded 6 hours. We suggest that TDx can be used to identify outliers within a health system, and structured chart reviews can identify key contributors to diagnostic delay. With more data, it is possible that a different cutoff (e.g., 12 hours) is a more reliable marker of the prolonged diagnosis, than 6 hours. In our cohort, 3 patients that were misdiagnosed with pneumonia/sepsis on admission, had TDx of greater than 12 hours (Table 3).

The present-on-admission and hospital-acquired indicators are used to analyze hospital performance. However, administrative coding of hospital-acquired venous thromboembolism is inaccurate [36]. TDx was successfully used to distinguish between PE at time of admission and PE acquired during hospitalization that we confirmed with subsequent chart reviews.

TTx can be used to further distinguish between patients with PE who have received timely treatment and those that have not. The combination of prolonged TDx and TTx is an optimal trigger for patients’ selection for structured chart review. Another group of patients to consider for review are those who have late diagnosis, but timely treatment. A high probability of PE is often a reason for initiation of the treatment before the PE diagnosis is confirmed. However, these patients might have a potential risk of overtreatment. A composite measure using TDx and TTx could be developed to reliably identify patients with poor diagnostic performance (late diagnosis and treatment) and distinguish between patients exposed to potential harm from overdiagnosis or late treatment.

### Strengths

To the best of our knowledge, this is the first study to assess time to diagnostic certainty using this approach to evaluate variability in diagnostic performance of a health system and identify patient outliers for structured chart review focused on the identification of the underlying contributors to diagnostic error or delay. Furthermore, we worked with critical care experts to determine the definitions for our study. The physician researchers who reviewed the EHR have extensive experience in conducting this type of research [23]. We scrutinized the study reliability and the Kappa coefficient was almost perfect.

### Limitations

This was a descriptive study using retrospective data from a single healthcare system that may limit the generalizability of our findings. We had a small sample size and wanted to use this study as a proof of concept for diagnostic certainty. The smaller number of patients allowed the physician researchers to closely examine the EHR while keeping the process and timeline feasible. This number of patients is typically used for studies like these as well as in the validation studies of EHR data [37]. The process of making a diagnosis depends on clinician expertise as well as access to diagnostic tool such as CT PA. Some centers may use other methods to diagnose a PE. Despite the limitations of using only CT PA as the TDx test, the reliability and ease of use justify our focus on this to the exclusion of other measures. Propensity scores to assess patients at risk, such as PE severity index and Wells score, were not used to describe baseline characteristics due to inconsistency of data. The sensitivity of prediction rules ranges from 49% to 65%, the specificity varies from 70% to 80% [38]. Although the Wells score is associated with diagnosis of PE, it has been shown to be more accurate in excluding patients without PE, rather than diagnosing those with PE. It has been shown that adherence to those rules by the providers was only in 35% of cases [39]. It is unknown if inconsistent usage of these prediction rules may have contributed to diagnostic delays in our cohort of patients. There could be situations when actual time of the first presentation was missed as we relied on clinical documentation. We did not account for the time of symptom onset. Therefore, the time distribution data was potentially skewed to the left and time TDx for PE might be underestimated. The rationale for not including these data was a high variability of time when symptoms occur and time when patients seek medical care. Additionally, TDx measure is intended to be used at the hospital-facility level rather than in primary care settings.

### Applications

The purpose of the study was to test the feasibility of using data that is easily abstracted from the health record to describe variability in TDx and TTx of a potentially life-threatening condition, saddle PE, in a health system. The usefulness of this search strategy to identify outliers for chart review is presented in this paper. Other applications not examined in this paper include the use of TDx as a trigger for root cause analysis and systems learning; time series monitoring of diagnostic performance and quality within a single institution; comparison of diagnostic performance for single conditions across diverse health care settings; and the application of the principle of time to diagnostic certainty to other conditions. While these applications have not been explored, it is the authors opinion that TDx may be a useful indicator of variability in diagnostic performance and an important additional tool to direct safety and quality enquiries.

### Future implication

The study findings support automate calculation of TDx and TTx as a subject for future inquiry. We are intended to apply electronic measure to a large cohort of PE, excluding isolated subsegmental PE, to incorporate all clinically relevant diagnoses. A sample chart review will be performed to determine the reliability of the measure to identify patients at risk of harm from over diagnosis or late treatment. An automated score and the structured chart review methodology, if determined valid and reliable in a subsequent study, could help a health system identify underlying contributors to its diagnostic performance. Finally, the deployment of the developed measure across different health system settings will allow us to determine its ability to discriminate between high and low diagnostic performers.

## Conclusion

We developed and successfully tested the concept of TDx for PE. We demonstrated that TDx allows reviewers to highlight variability in diagnostic performance of a health system; and identify patient outliers for structured chart review focused on the identification of the underlying contributors to diagnostic error or delay. Time to diagnostic certainty along with time to treatment may be useful as a composite measure of PE to be implemented and used to assess diagnostic performance and quality of care at the hospital-facility level.
